# Pregnant Woman in Outcomes with Prosthetic Heart Valves

**DOI:** 10.3390/jcdd11110353

**Published:** 2024-11-04

**Authors:** Giunai Sefiyeva, Ulyana Shadrina, Tatiana Vavilova, Olga Sirotkina, Andrey Bautin, Aigul Chynybekova, Anna Pozhidaeva, Ekaterina Stepanovykh, Anna Starshinova, Dmitry Kudlay, Olga Irtyuga

**Affiliations:** 1 Almazov National Medical Research Centre, St. Petersburg 197341, Russia; namexxx555@mail.ru (G.S.); shadrina_um@almazovcentre.ru (U.S.); vtv.lab.spb@mail.ru (T.V.); olga_sirotkina@mail.ru (O.S.); abautin@mail.ru (A.B.); chynybekova11@mail.ru (A.C.); pozhidaeva_am@almazovcentre.ru (A.P.); stepanovykh_ed@almazovcentre.ru (E.S.); olgir@yandex.ru (O.I.); 2 Department of Pharmacognosy and Industrial Pharmacy, Faculty of Fundamental Medicine, Lomonosov Moscow State University, Moscow 119991, Russia; d624254@gmail.com; 3 FMBA Institute of Immunology, Moscow 115478, Russia; 4 Department of Pharmacology, Institute of Pharmacy, I.M. Sechenov First Moscow State Medical University, Moscow 119991, Russia

**Keywords:** pregnant woman, prosthetic heart valves, mechanical valve prostheses, biological valve prosthesis, anti-factor Xa

## Abstract

We here sought to assess thrombotic and hemorrhagic complications and associated risk factors during pregnancy, delivery, and postpartum in women with prosthetic heart valves (PHV). Methods: The retrospective cohort study covered January 2011 to December 2022. The objective of the study was to assess the risk factors and frequency of thrombotic and hemorrhagic complications during pregnancy, delivery, and the postpartum period in women with PHV based on the experience of one perinatal center. We included 88 pregnancies with 77 prosthetic heart valves (PHV), which were divided into two groups, mechanical valve prostheses (MVP) (n = 64) and biological valve prosthesis (BVP) (n = 24). In the study we analyzed pregnancy outcomes, as well as thrombotic and hemorrhagic complication frequencies. Results: Of 88 pregnancies, 79 resulted in live births. In the MVP group, there were six miscarriages (9.4%) and two medical abortions (3.1%), including one due to Warfarin’s teratogenic effects. No miscarriages were reported in the BVP group, but one fetal mortality case (4.2%) occurred. During pregnancy, 11 MVP cases (17.2%) experienced thrombotic complications. In the BVP group, one patient (4.2%) had transient ischemic attack (TIA). Two MVP cases required surgical valve repair during pregnancy, and one in the post-delivery stage was caused by thrombotic complications. Postpartum, two MVP cases had strokes, and in one MVP patient, pulmonary embolism was registered, while no thrombotic complications occurred in the BVP group. Hemorrhagic complications affected 15 MVP cases (17.9%) in the postpartum period. There were no registered cases of maternal mortality. Conclusions: The effective control of anti-factor Xa activity reduced thrombotic events. However, the persistently high incidence of postpartum hemorrhagic complications suggests a need to reassess anticoagulant therapy regimens, lower target levels of anti-Xa, and reduce INR levels for discontinuing heparin bridge therapy. Despite the heightened mortality risk in MVP patients, our study cohort did not have any mortality cases, which contrasts with findings from other registries.

## 1. Introduction

Advancements in cardiac surgery have increased the frequency of pregnancies among women with prosthetic heart valves (PHV). Patients with mechanical valve prostheses (MVP) are at significantly higher risks of complications during pregnancy, delivery, and the postpartum period, primarily due to the necessity of maintaining anticoagulant therapy while avoiding hemorrhagic complications.

Pregnancy is a prothrombotic state, where the combination of venous stasis and hypercoagulability results in nearly a 5-fold increase in the risk of venous thromboembolism [1]. Thromboembolic events in these patients may be linked to sub-therapeutic anticoagulation during the switch in anticoagulant therapy, especially in the first trimester, or the insufficiency of low-molecular-weight heparins (LMWHs). Mechanical valve thrombosis is the most dreaded complication, carrying a 20% risk of mortality [2].

While vitamin K antagonists (VKAs), with Warfarin being the most widely used, are highly effective medications for preventing mechanical valve thrombosis, they also pose risks to the fetus. VKAs cross the placenta and have a dose-dependent relationship with adverse outcomes, such as miscarriage, stillbirth, and embryopathy, occurring predominantly at doses exceeding 5 mg daily [3]. The continued use of VKAs is associated with an elevated risk of fetal hemorrhage during vaginal delivery [1].

Unlike VKAs, LMWHs do not cross the placenta and are not associated with congenital malformations. However, using LMWH requires dose adjustments depending on anti-factor Xa activity due to changes in renal clearance and the volume of distribution during pregnancy [4]. The correct anticoagulation therapy for pregnant women with MVP to reduce the risk of maternal and fetal complications presents a challenging dilemma. Currently, three regimens of anticoagulation are followed globally [5,6]:

1. Warfarin throughout pregnancy with unfractionated heparin (UFH) near-term;
2. Switching from Warfarin to UFH or LMWH between 6 and 12 weeks of gestation and near-term;
3. LMWH throughout pregnancy.

The National Russian Guidelines allow delivery 12 h after the last dose of LMWH [7], while the European Guidelines require a mandatory switch to UFH 36 h before the planned delivery. Women with valvular heart disease (VHD) who are pregnant or considering pregnancy should be managed by a multidisciplinary pregnancy team, including a cardiologist, obstetrician and hematologist in some cases.

The objective of this study was to assess the risk factors and frequency of thrombotic and hemorrhagic complications during pregnancy, delivery, and the postpartum period in women with PHV based on the experience of one perinatal center.

## 2. Material and Methods

### 2.1. Patient Characteristics

**Figure 1:**
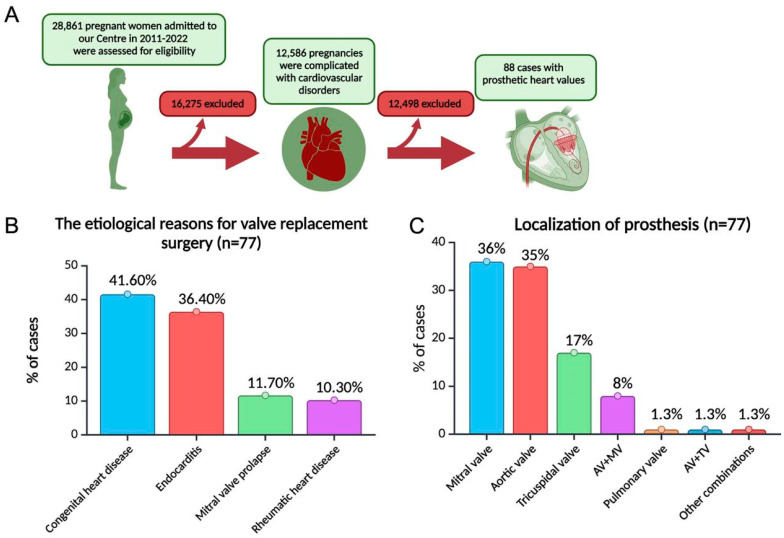
Characterization of the studied patient cohort. (**A**) Patient recruitment process. (**B**) The etiological reasons for valve replacement surgery. Total number of observations n = 77. (**C**) Localization of prosthesis. Total number of observations n = 77. AV—aortic valve, MV—mitral valve, TV—tricuspid valve (Created with BioRender.com).

The retrospective cohort study spanned from January 2011 to December 2022, during which 28,861 patients were admitted to the specialized perinatal center of Almazov National Medical Research Center. Among these, 12,586 women were diagnosed with cardiovascular diseases (Figure 1A).

Pregnant women with PHV were retrospectively enrolled in the study. Notably, our medical center, being a federal institution, caters to patients from all regions of Russia, and our multidisciplinary team does not always observe them from the first trimester of pregnancy. All participants signed informed consent prior to their inclusion in the study.

The study comprised 88 cases of pregnancy in 77 patients with PHV, with 10 patients undergoing observation twice or more times because of subsequent pregnancies. Patients were categorized into two groups based on the type of prosthesis: the first group included 64 pregnancies in patients with MVP, while the second group included 24 pregnancies in women with biological valve prosthesis (BVP).

The study encompassed 88 pregnancy events, with 73% (n = 64) involving women with MVP and 27% (n = 24) involving patients with BVP. Valve replacement occurred primarily due to primary infectious endocarditis (36.4%), chronic rheumatic heart disease (10.3%), mitral valve prolapse (11.7%) and congenital heart diseases (41.6%) (bicuspid aortic valve, subvalvular aortic stenosis, patent ductus arteriosus (Figure 1B)). The mitral valve was the most common valve to be replaced (Figure 1C).

The baseline characteristics of all patients are detailed in Table 1, and the characteristics of the patients with thrombotic complications during pregnancy are presented in Supplementary Table S1. Maternal mortality was defined as death during pregnancy or within 42 days of pregnancy resolution, related to or aggravated by the pregnancy or its management, excluding unintentional or accidental causes [8]. Miscarriage was defined as pregnancy loss up to 22 weeks, stillbirth as fetal loss beyond 22 weeks, and live births as pregnancies resulting in a live neonate. Thrombotic events encompassed valve thrombosis, pulmonary embolism, deep vein thrombosis, or any ischemic cardiovascular or cerebrovascular events. Hemorrhagic complications included major bleeding, defined as a hemorrhage resulting in at least a 1-g/dL (or 0.62-mmol/L) decrease in hemoglobin, the need for blood product transfusion, or end-organ damage such as hemorrhagic cerebrovascular accident or retinal bleeding, postpartum hemorrhage defined as increased blood loss (>500 mL after vaginal delivery or >1000 mL after cesarean delivery) directly after delivery and up to 24 h postpartum [9] hematometra, vulvovaginal hematomas, surgical area hematomas, hemoperitoneum, with the significance of bleeding assessed based on the need for surgical treatment. Additionally, the study evaluated complications such as arrhythmias requiring treatment, worsening heart failure, and endocarditis.

### 2.2. Methods

Echocardiography (Vivid 7, GE, Philadelphia, PA, USA) was conducted at least 3 times during pregnancy for all participants. Transesophageal echocardiography was performed at any time of pregnancy in the case of suspected dysfunction or thrombosis of prosthetic valves. The N-terminal brain natriuretic propeptide (NT-proBNP) concentration was determined using the quantitative electrochemiluminescence immunoassay technique Elecsys proBNP II test) using a Cobas E 411 analyzer (Roche, Mannheim, Germany). Until 2016, the level of anti-factor Xa activity was not monitored in this category of patients in the Almazov Centre.

Since 2016, anti-factor Xa activity has been monitored using chromogenic assays (ACL TOP CTS 500, Instrumentation Laboratory, Bedford, MA, USA). Target anti-Xa levels were set at 0.8–1.0 IU/mL (for aortic position) and 1.0–1.2 IU/mL (for mitral and tricuspid valve prostheses) [1,10,11,12]. For patients on LMWH, anti-Xa levels were monitored daily until target values were reached and at least once every 7 days thereafter.

International normalized ratio (INR) was determined in all MVP patients. Target INR levels were 2.5–3.5 for mitral and tricuspid positions and 2.0–3.0 for aortic position. Higher anticoagulation levels with INR of 2.5–3.5 were achieved in patients with additional risk factors for thrombosis (medium/high thrombogenicity of the prosthesis and/or the history of thromboembolism, atrial fibrillation, mitral stenosis of any degree). INR monitoring was conducted daily until target values were reached and at least once a week thereafter.

Statistical analysis was performed using STATISTICA v. 10.0 (StatSoft Inc., Tulsa, OK, USA). The normality of data distribution was evaluated by the Shapiro–Wilk normality test. Our data were not-normally distributed. The Mann–Whitney U-test was applied for the comparison of quantitative indicators. Chi-Square or Fisher’s exact test were utilized to compare categorical data. Data are presented as median and interquartile ranges (IQR). Statistical significance was determined at *p*-values of ≤0.05.

## 3. Results

All patients with MVP were receiving different anticoagulant therapy regimens corresponding to current guidelines, as follows: either VKA throughout pregnancy, either switching from VKA to LMWH in the first trimester of pregnancy, or either LMWH during pregnancy.

The median maternal age was 31 (IQR, 28–34) and the gestational age at delivery was 38 (IQR, 37–38) weeks. Notably, 38% of patients did not manifest clinical signs of heart failure before pregnancy, while others exhibited chronic heart failure not exceeding NYHA class II. However, during pregnancy, some patients experienced an increase in NYHA class up to class III—3.4% (n = 3) during the first, 4.87% (n = 4) during the second, and 11.4% (n = 9) during the third trimester (Table 1).

The median NT-proBNP concentration during pregnancy was 202 (IQR, 71–443) pg/mL in patients with MVP and 123 (IQR, 63–180) pg/mL in patients with BVP. In the postpartum period, heart failure worsening was generally not observed, except for in two cases (2.53%) where chronic heart failure escalated from NYHA class I to IV due to prosthesis thrombosis and systemic infection. The NT-proBNP concentration in the postpartum period was 415 (IQR, 204–826) pg/mL for MVP patients and 132.5 (IQR, 83–256) pg/mL for BVP patients (Table 1).

Cardiac arrhythmias, particularly ventricular extrasystoles, were the most prevalent complications in both groups—40.6% in MVP patients and 41.7% in BVP patients (*p* = 0.930) (Table 1). Nonsustained paroxysmal ventricular tachycardia occurred in 9.4% of MVP patients and 4.17% of BVP patients (*p* = 0.421) (Table 1). Sustained ventricular tachycardia was observed in one MVP case (1.56%), with none reported in the BVP group (*p* = 0.538) (Table 1).

Of the observed pregnancies, 79 concluded with live births. In the MVP group receiving Warfarin therapy, there were six cases (9.4%) of miscarriages and two cases (3.1%) of medical abortion, with one due to teratogenic effects of Warfarin dose > 5 mg/day. No miscarriages or therapeutic abortions were reported in the BVP group, but one case (4.17%) of fetal mortality at 23 weeks was documented (*p* = 0.538) (Table 1).

### 3.1. Type of Delivery

**Figure 2:**
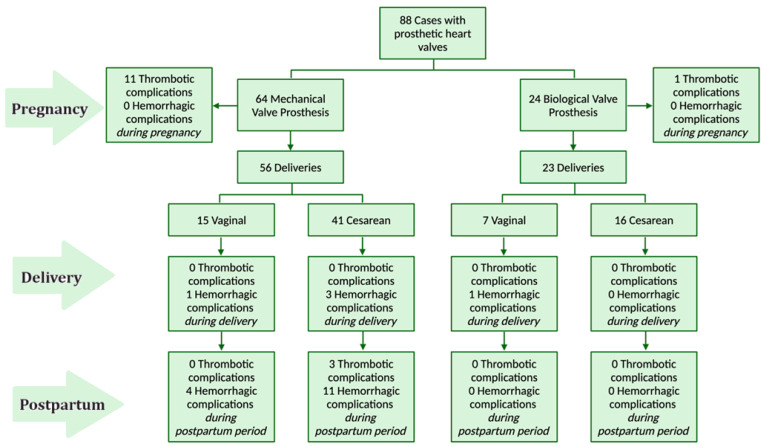
Complications in pregnancies with mechanical valve prosthesis and biological valve prosthesis (Created with BioRender.com).

Patients in both groups predominantly underwent cesarean delivery, with 73.2% (n = 41) for MVP patients and 69.6% (n = 16) for BVP patients (Figure 2). Vaginal deliveries were recorded in 26.8% (n = 15) of MVP cases and 30.4% (n = 7) of BVP cases.

### 3.2. Thrombotic Complications

In the MVP group, 17% (n = 11) of patients had thrombotic complications, including 10 cases (15.6%) of prosthetic valve thrombosis and 1 case of transient ischemic attack (TIA). No prosthetic valve thrombosis was reported in the BVP group (*p* = 0.0397), but one patient (4.17%) had TIA in the third trimester (Figure 2, Table 1). Prosthetic valve thrombosis predominantly occurred in the mitral (50%) and aortic (40%) positions. In all cases of prosthetic valve thrombosis, the causal factors were either patients discontinuing anticoagulant therapy or a failure to regulate anti-Xa activity during the administration of LMWH. Despite thrombotic complications, only 2 out of 11 cases required surgical valve repair during pregnancy and one after delivery. In all other pregnant patients who were receiving LMWH, the target anti-Xa levels were achieved, at 0.8–1.0 IU/mL for aortic position and 1.0–1.2 IU/mL for mitral and tricuspid valve prostheses. In the postpartum period, two cases of stroke and one case of pulmonary embolism were registered in the MVP group, with no thrombotic complications in the BVP group (*p* = 0.629) (Figure 2).

### 3.3. Hemorrhagic Complications

During cesarean delivery, 7% (n = 3) of MVP cases experienced hemorrhagic complications, while no such cases were reported in the BVP group (*p* = 0.266) (Figure 2). Hemorrhagic complications during vaginal delivery were minimal, with one case (7%) in MVP patients and one case (14.3%) in BVP patients (Figure 2). In the early postpartum period, 17.86% (n = 10) of MVP cases had bleeding with hematoma formation requiring surgical intervention and blood transfusions, while no hemorrhagic complications were noted in the BVP group (*p* = 0.021). One case of bleeding after medical abortion and five cases of hematoma with minor bleeding in the first month after delivery were reported in MVP patients. Hemorrhagic complications typically occurred on the fifth day (IQR, 4–6) of the postpartum period. Despite frequent complications, no fatal outcomes were reported during pregnancy or in the postpartum period.

## 4. Discussion

In the retrospective cohort study that included 88 pregnancies, patients with MVP had more frequent complications compared to those with BVP. While thrombotic events occurred throughout the entire pregnancy and in postpartum period, bleeding complications were registered only in delivery and postpartum periods.

Pregnancy in women with MVP poses a considerable risk of complications, including valve thrombosis, thromboembolism, and bleeding (WHO risk class III) [1]. Despite this, there is no agreement among physicians on the optimal anticoagulation regimen, particularly during the first trimester [2]. Furthermore, guidelines lack consensus on the treatment protocol for Warfarin-induced major hemorrhage in pregnant patients with MVP, with studies primarily consisting of case presentations [13].

The Observational Registry of Pregnant Women with Cardiovascular Pathology (ROPAC) indicates that 43% of patients with VHD had rheumatic heart disease [13]. However, our study revealed congenital heart disease as the primary reason for valve replacement, which is consistent with findings reported in the National Register of Denmark [4]. The localization of valve prostheses was also comparable between our study and the Danish registry, with prosthetic aortic valve (PAV) frequency at 35% and prosthetic mitral valve (PMV) at 36% in our study, compared to 42% and 25%, respectively, in the Danish registry [4].

According to the results of the ROPAC registry, in developing countries, cesarean deliveries were performed in 64.7% of PHV patients and in 46.6% of cases in the general population of women in labor [13]. Our study shows a high rate of cesarean deliveries, at 73.2% in MVP patients and 69.6% in BVP patients, exceeding available European data. We found only one fetal loss (1.13%) in the BPV group, which is significantly less compared to the literature [5,14,15,16] frequencies varying from 24% to 65%.

Our study has documented a high incidence of thromboembolic events, affecting 17% of patients with MVP, which falls within the frequency range of 1.4% to 19.6% reported in the literature [17]; however, these values are less than in the study of Popelova et al., where prosthetic valve thrombosis occurred in 26% of patients [18]. We found that thrombosis occurred in both mitral and aortic positions, in contrast to the findings of Vause et al., where prosthetic thrombosis complicated the pregnancy only in women with MVP in the mitral position [9]. The ROPAC registry reports a 7% prosthetic thrombosis incidence among MVP patients, with 18% of these patients having a lethal outcome [13], and other studies [19] documented even higher risks of thromboembolic events, ranging from 7% to 23%, with mortality reaching 40%. No cases of maternal death were observed in our series.

Increased cardiac output during pregnancy and heightened coagulability contribute to the added risk of mechanical valve thrombosis in women with PHV [17,20,21,22]. The elevated levels of circulating pro-coagulant factors and maternal hormones lead to a decrease in prothrombin time, activated partial thromboplastin time, thrombin time, and INR [23,24]. Therefore, achieving adequate anticoagulation during pregnancy is challenging, which in turn elevates the risk of thromboembolic events and mechanical valve thrombosis.

The issue of anticoagulant therapy in pregnant patients with PHV remains relevant, given Warfarin’s teratogenic effects and heparin’s lesser efficacy compared to Warfarin in preventing thrombotic events (10% vs. 3.9%) [5,25,26,27]. During the first trimester, heparin does not offer a safer alternative to Warfarin [17], while Warfarin is contraindicated in the first trimester due to its teratogenic potential [18,28]. For that reason, despite Warfarin being the most effective anticoagulant, most studies have switched to using LMWHs or UFHs as they do not cross the placenta and do not cause birth defects [29,30]. On the other hand, there is evidence suggesting that VKAs are better than LMWHs or UFHs at preventing prosthetic valve thrombosis in the first trimester, and there is no statistical difference in the rate of spontaneous abortion between the two regimens [18]. Meanwhile, some reports suggest that using Warfarin during pregnancy is relatively safe if adequate anticoagulation can be achieved with doses of 5 mg or less [1,31]. A systematic review and meta-analysis by Hassouna et al. concluded that using limited doses of Warfarin (≤5 mg/day) throughout pregnancy could improve fetal outcomes without risking maternal safety [32]. They found that patients receiving more than 5 mg/day of Warfarin were more prone to bleeding complications and miscarriage. Khamoushi et al. also reported that using low-dose Warfarin (≤5 mg/day) during pregnancy is generally safe, with minimal complications for both the mother and fetus [33]. In our study, patients used all three anticoagulant therapy regimens, including taking warfarin at a dose not exceeding 5 mg/day in the first trimester. We registered birth defects only in one patient who received 5 mg of Warfarin in the first trimester, while the rate of miscarriage, mostly in patients who did not stop taking VKAs in the first trimester, was quite high.

The high rate of thrombotic complications in our study groups was mainly due to patients not following through with anticoagulant therapy and stopping it on their own. Another significant reason for thrombotic complications in the study was the lack of control over anti-Xa level before hospitalization, resulting in subpar doses of prescribed LMWHs. Many studies that described thrombosis cases in patients receiving LMWHs also noted that these occurred either without control over anti-Xa activity or at subtherapeutic levels of anti-Xa [34,35]. Our study has confirmed the effectiveness of LMWH dose adjustments alongside regular assessments of anti-Xa level in achieving target levels corresponding to the existing national recommendations for the management of pregnant women [1,7]. By controlling anti-factor Xa activity and reaching the target values, we did not register any cases of prosthetic valve thrombosis, the most drastic complication found in this patient group.

In the postpartum period, the major complication in our patient cohort was bleeding, occurring in more than 20% of cases, with 17% requiring relaparotomy. Other studies [5,14,15,16,36,37] reported hemorrhage rates varying between 6% and 23%. According to the ROPAC registry [13], bleeding was recorded in 5.1% of BVP cases and in 4.9% of cases in the general population of women in labor. In our study, however, we did not register hemorrhagic complications in BVP patients during pregnancy or in the postpartum period. During delivery, there was only one case (4.17%) of hemorrhagic complications, which did not require surgical intervention.

Approaches to the prescription of anticoagulant therapy may be adjusted to account for COVID-19, which is associated with the effect of infection on myocardial tissue and on thrombus formation [38,39].

In contrast to thrombosis, hemorrhagic complications, both in our study and according to the ROPAC registry [13], were not associated with worsening heart failure, maternal deaths, or fetal loss. In our study, we did not record any cases of maternal death despite the previous results showing a 1.4% maternal mortality rate in MVP patients and 1.5% in BVP patients [28]. However, given the high rates of severe and potentially life-threatening complications and high rates of miscarriage, these women require caution in counseling, and close observation with supervision is recommended when pregnancy occurs.

An extensive experience managing patients with cardiovascular conditions increased our ability to adjust for adverse pregnancy outcomes, and the implications of our findings could meaningfully affect clinical practice.

## 5. Limitations

This study has limitations, such as the small number of patients included in this study (less than 100 patients). Also, another limitation of this study is that it offers retrospective data, and not all the patients were managed at this center in the first trimester.

## 6. Conclusions

Women with MVP face an elevated risk of maternal morbidity, particularly blood clotting and bleeding complications during pregnancy. The increased rate of thrombotic events in these patients results from the insufficient monitoring of anti-Xa level and patients discontinuing anticoagulant therapy on their own.

The persistent high incidence of hemorrhagic complications in the postpartum period, requiring relaparotomies, demands a revision of anticoagulant therapy regimens in the postpartum period [40] and the possible lowering of INR levels to discontinue heparin bridge therapy.

Despite the heightened mortality risk in MVP patients, our study cohort, overseen by a multidisciplinary team, did not have any mortality cases, in contrast to findings from other registries. Therefore, it is crucial for women with MVP to be thoroughly informed about the potential risks associated with pregnancy, and to receive comprehensive support and care throughout the entire pregnancy, childbirth, and postpartum periods from a specialized multidisciplinary team.

## Figures and Tables

**Table 1:** Baseline characteristics of the patients with mechanical valve prosthesis (MVP) and biological valve prosthesis (BVP) before and during pregnancy.

Patient Characteristic	Patients with MVP (n = 64)	Patients with BVP (n = 24)	*p*-Value MVP vs. BVP
Before Pregnancy			
Age, median (IQR), y	30.5 (27–34)	31.5 (30–33)	0.290
Current smoker, n (%)	4 (6.25)	2 (8.33)	0.730
Pre-existing hypertension, n (%)	4 (6.25)	2 (8.33)	0.730
Arrhythmia, n (%)	23 (35.94)	11 (45.83)	0.396
Previous atrial fibrillation, n (%)	6 (9.38)	4 (16.7)	0.337
NYHA functional class, n (%)			
I	21 (32.81)	8 (33.3)	0.963
II	24 (37.5)	4 (16.7)	0.062
III–IV	0	0	NA
Previous medication, n (%)			
β-Blockers	16 (25)	2 (8.33)	0.034
ACE inhibitor	3 (4.69)	1 (4.16)	0.917
Diuretics	3 (4.69)	0	0.280
During Pregnancy			
Maternal hospital admission (any reasons), n (%)	48 (75)	13 (54.2)	0.059
Maternal hospital admission for cardiac reason, n (%)	43 (67.2)	8 (33.3)	0.004
*Cardiac complications*			
NYHA functional class increasing during first trimester of pregnancy, n (%)	Patients with MVP (n = 64)	Patients with BVP (n = 24)	
I	1 (1.56)	1 (4.17)	0.465
II	1 (1.56)	0	0.538
III–IV	0	0	NA
NYHA functional class increasing during second trimester of pregnancy, n (%)	Patients with MVP (n = 58)	Patients with BVP (n = 24)	
I	1 (1.72)	0	0.518
II	2 (3.44)	1 (4.17)	0.875
III–IV	0	0	NA
NYHA functional class increasing during third trimester of pregnancy, n (%)	Patients with MVP (n = 56)	Patients with BVP (n = 23)	
I	0	0	NA
II	5 (8.93)	2 (8.69)	0.974
III	1 (1.79)	1 (4.34)	0.510
IV	0	0	
NT-pro-BNP during pregnancy, median (IQR), pg/mL	202 (71–443)	123 (63–180)	0.150
Non-sustained ventricular tachycardia, n (%)	6 (9.4)	1 (4.17)	0.421
Sustained ventricular tachycardia, n (%)	1 (1.56)	0	0.538
Ventricular extrasystole, n (%)	26 (40.6)	10 (41.7)	0.930
Atrial fibrillation, n (%)	1 (1.56)	0	0.538
Prosthetic valve thrombosis, n (%)	10 (15.6)	0	0.0397
Ischemic cerebrovascular accident, n (%)	1 (1.56)	1 (4.17)	0.465
Pulmonary embolism	0	0	
Pregnancy-induced hypertension, n (%)	9 (14)	1 (4.17%)	0.193
Pre-eclampsia, n (%)	5 (7.81)	0	0.159
*Pregnancy outcomes*			
Live birth, n (%)	56 (87.5)	23 (98.9)	0.250
Pre-term delivery < 37 wk, n (%)	8 (14.3)	2 (8.7)	0.460
Miscarriage < 22 wk, n (%)	6 (9.38)	0	0.120
Therapeutic abortion: maternal condition, n (%)	1 (1.56)	0	0.538
Therapeutic abortion: fetal abnormalities, n (%)	1 (1.56)	0	0.538
Pregnancy duration, median (IQR), wk	38 (37–38)	38 (37–39)	0.104
Birth weight, median (IQR), g	3035 (2540–3275)	2970 (2700–3240)	0.966
Apgar score < 7/10, n (%)	9 (16)	2 (8.7)	0.390
Fetal mortality, n (%)	0	1 (4.17)	0.538

## Data Availability

All source data are in the Supplementary Files attached to the article; if you need clarifications, or need additional information, you can write to the email: olgir@yandex.ru.

## Supplementary Materials

The following supporting information can be downloaded at: https://www.mdpi.com/article/10.3390/jcdd11110353/s1, Table S1. Characteristics of the patients with thrombotic complications during pregnancy.
