# Supplementary material for: Pregnant Woman in Outcomes with Prosthetic Heart Valves
Source: J Cardiovasc Dev Dis. 2024 Nov 4;11(11):353. doi: 10.3390/jcdd11110353 (PMC11595173; doi:10.3390/jcdd11110353)
Supplement: Supplementary file 1 [file jcdd-11-00353-s001.zip › jcdd-3281277-supplementary.pdf]

Supplementary Table S1. Characteristics of the patients with thrombotic complications during pregnancy

**Patient**	**Valve position**	**Type of prosthesis**	**Week of AC switch**	**Anticoagulant therapy**					**Anti-Xa levels during thrombotic event**	**INR levels during event**	**Onset time of complications, wk**	**Thrombotic events during pregnancy**	**Pregnancy duration, wk**	**Pregnancy outcome**	**Surgery during pregnancy/after delivery**	**Type of delivery**	**Maternal Mortality**
				1st trimester	2nd trimester	3rd trimester	Before delivery	After delivery									
**1**	TV	MVP	6	LMWH	No	No	No	VKAs+LMWH	0.67	NA	8 4/7	PVT	10	Misscariage	Reprosthesis at 9 wk of pregnancy	NA	No
**2**	MV	MVP	35	VKAs	VKAs	VKAs +LMWH*	LMWH	VKAs UFH	NA	1	35	PVT	37	Live birth	Reprosthesis on 16 d after delivery	Cesarean	No
**3**	MV	MVP	4; 34	LMWH irregular intake	LMWH irregular intak	LMWH*+ VKAs	LMWH	VKAs UFH	NA	2.18	34	PVT	35	Live birth	Conservative therapy Regression of thrombosis	Cesarean	No

					e												
**4**	AV	MVP	9; 23	LMWH	VKAs + LMW H*	VKAs	VKAs	LMW H VKAs	NA	NA	23	PVT	37	Live birth	Conservative therapy Regression of thrombosis	Cesarean	No
**5**	AV	MVP	3; 15	LMWH	VKAs	VKAs	VKAs	VKAs UFH	NA	1.68	15	PVT	37	Live birth	Thrombectomy at 15 wk of pregnancy	Cesarean	No
**6**	MV	MVP	6	LMWH	LMW H	LMWH	LMW H	LMW H VKAs	NA	NA	6	TIA	37	Live birth	No	Cesarean	No
**7**	MV	MVP	4; 12	LMWH	LMW H	VKAs	LMW H	LMW H VKAs	NA	1.15	23	PVT	34	Live birth	Conservative therapy Regression of thrombosis	Cesarean	No

**8**	MV	MVP	3	LMWH	LMWH	LMWH	LMWH	LMW VKAs	0.7	NA	24	PVT	32	Live birth	Conservative therapy Regression of thrombosis at 28 wk of pregnancy	Vaginal	No
**9**	MV	MVP	3	LMWH	LMWH	LMWH	LMWH	VKAs UFH	0.55	NA	12	PVT	27	Live birth	Conservative therapy Regression of thrombosis at 19 wk of pregnancy	Cesarean	No
**10**	AV	MVP	3; 18; 36	LMWH	VKAs + LMWH*	VKAs	LMWT	VKAs UFH	NA	NA	18	PVT	38	Live birth	Conservative therapy Regression of thrombosis in 2nd trimester of pregnancy	Vaginal	No
**11**	AV	MVP	5; 12	LMWH	VKAs	VKAs	VKAs	VKAs UFH	NA	1.6	NA	PVT	36	Live birth	Conservative therapy Regression of thrombosis in 2nd trimester of pregnancy	Cesarean	No
**12**	AV	BVP	NA	LMWH from 12 wk	LMWH	LMWH	LMWH	LMWH	NA	NA	12	TIA	38	Live birth	Conservative therapy	Vaginal	No

AC–anticoagulants therapy, AV–aortic valve, BVP–biological valve prosthesis, LMWH–low molecular weight heparin, MV–mitral valve, MVP–mechanical valve prosthesis, NA–no data, PVT–Prosthetic valve thrombosis, TIA–transient ischemic attack, TV–tricuspid valve, VKAs–Vitamin K antagonist, UFH–unfractionated heparin *- Adding LMWH when the target INR levels have not been reached
